# Multimerin-1 and cancer: a review

**DOI:** 10.1042/BSR20211248

**Published:** 2022-02-25

**Authors:** Mareike G. Posner

**Affiliations:** Department of Life Sciences, Manchester Metropolitan University, Manchester, United Kingdom

**Keywords:** biomarkers, cancer, molecular basis of health and disease, transcription

## Abstract

Multimerin-1 (MMRN1) is a platelet protein with a role in haemostasis and coagulation. It is also present in endothelial cells (ECs) and the extracellular matrix (ECM), where it may be involved in cell adhesion, but its molecular functions and protein–protein interactions in these cellular locations have not been studied in detail yet. In recent years, MMRN1 has been identified as a differentially expressed gene (DEG) in various cancers and it has been proposed as a possible cancer biomarker. Some evidence suggest that MMRN1 expression is regulated by methylation, protein interactions, and non-coding RNAs (ncRNAs) in different cancers. This raises the questions if a functional role of MMRN1 is being targeted during cancer development, and if MMRN1’s differential expression pattern correlates with cancer progression. As a result, it is timely to review the current state of what is known about MMRN1 to help inform future research into MMRN1’s molecular mechanisms in cancer.

## Background

**Figure 1:**
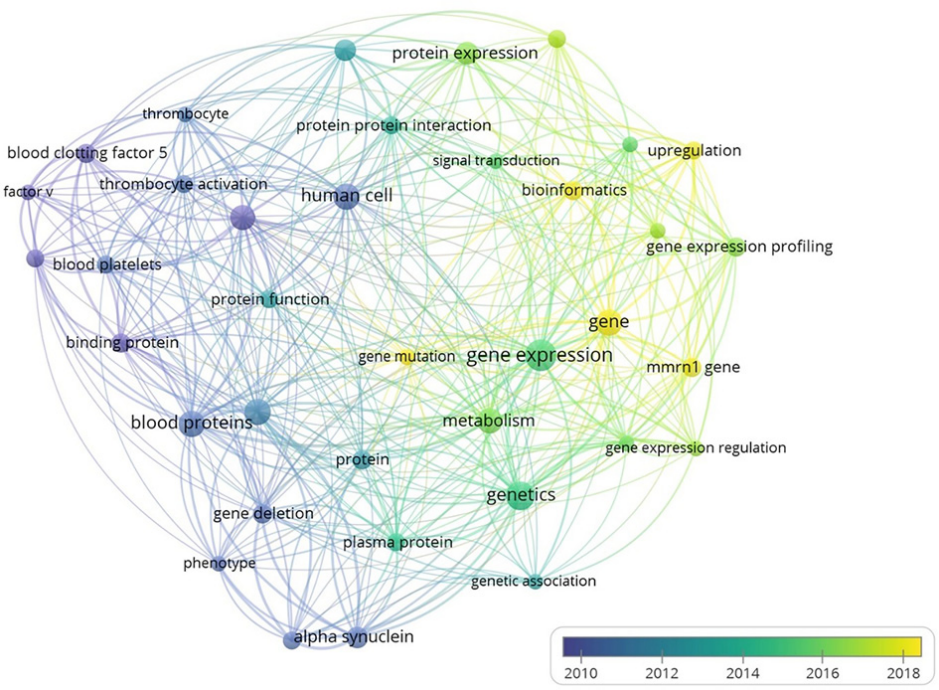
Bibliometric network analysis of MMRN1 in the scientific literatures The network analysis was carried out using VosViewer (https://www.vosviewer.com, [202]). Over time, the research focus has shifted from MMRN1’s role in platelets (purple, blue) to its differential gene expression (green, yellow) in recent years.

Multimerin-1 (MMRN1) is a member of the EMILIN/multimerin family of proteins, found in platelets (α-granules of resting platelets), megakaryocytes, endothelial cells (ECs, Weibel–Palade bodies) and the extracellular matrix (ECM) [1–6]. In response to specific triggers, MMRN1 is secreted from platelet α-granules and Weibel–Palade bodies of ECs [7–9]. MMRN1 platelet-related functions include platelet adhesion [5,10–13], factor V regulation [14–18], and MMRN1 deficiency is associated with bleeding risks in Quebec platelet disorder [19–21]. These functions, however, shall not be covered in the current article as these have already been reviewed [22]. Instead, the focus will be on MMRN1’s non-platelet-related functions, including *mmrn1* differential gene expression and its proposed use as a cancer biomarker [23–25]. A bibliometric network analysis illustrates how the research on MMRN1’s platelet-related functions i.e., ‘blood’, ‘platelets’, ‘factor V’, has shifted to the analysis of MMRN1 ‘gene expression’, ‘protein expression’, using ‘bioinformatics’ over recent years (Figure 1). The reports on MMRN1’s differential gene expression and also highlight the lack of molecular studies aimed to describe MMRN1’s physiological functions.

Whereas the role of other EMILIN/multimerin family members in cancer has been the focus of various studies [26–37], a molecular mechanism for MMRN1 is yet to be described. It is also unclear if MMRN1’s functions are indiscriminatory of its cellular locations, platelets, ECs and the ECM, which all play roles in cancer. Platelets are associated with metastasis [38–40]. The interaction with platelets, facilitates circulating cancer cells to evade natural killer cells and adhere to vascular walls. The cancer cells can subsequently cross the vascular endothelium and exit the circulation (extravasation), which can lead to metastasis [39–41]. The recruitment of the ECM and granulocytes by activated platelets drives the formation of the early metastatic niche that allows the cancer to survive and proliferate [39]. Activated platelets also release pro-angiogenic growth factors (e.g., vascular endothelial growth factor (VEGF)) which regulate tumour angiogenesis [41]. EC migration and proliferation are activated by VEGF [42] and interaction between ECs and cancer cells can further promote angiogenesis [43]. The ECM is involved in various processes including epithelial-to-mesenchymal transition and metastasis [44,45], the latter being an example of cancer plasticity [46]. The tumour ECM, which is mainly derived from tumour-associated fibroblasts, is denser and stiffer and has an altered, tumour-specific molecular expression profile, which alters cell–ECM interactions and signalling cascades to support tumour growth. Cancer plasticity is associated with changes in gene expression patterns, which is why MMRN1’s differential expression pattern in cancer is of great interest.

MMRN1 expression is significantly downregulated in 17 (bladder, breast, colon, oesophagous, liver, lung (adenocarcinoma and squamous cell carcinoma), ovary, prostate, rectum, renal (cell carcinoma and papillary cell carcinoma), skin, stomach, testis, thyroid, uterus (uterine carcinosarcoma and uterine corpus endometrial carcinoma)) out of 22 cancer types, and significantly upregulated in acute myeloid leukaemia and pancreatic cancer (TNM plot pan-cancer analysis [47]). MMRN1 is also expressed by various cancer cell lines. Despite such analyses and clinical observations of MMRN1 differential expression in cancer [24,25], the molecular mechanisms driving these changes are currently unknown and raises various questions: is MMRN1 differential expression merely a result of other cellular changes or is it regulated by tumour cells to aid the disease development and/or progression? How is MMRN1 expression regulated in cancer? What are MMRN1 functions in different cell types? Can MMRN1 expression levels help diagnose cancer and/or cancer stages? Does MMRN1 interact with tumour cells via protein–protein interactions? This review is intended to provide an overview of the state-of-the-art in this field, with the aim to provide information towards addressing these questions in future.

## MMRN1 protein–protein interactions and their possible role in cancer

**Figure 2:**
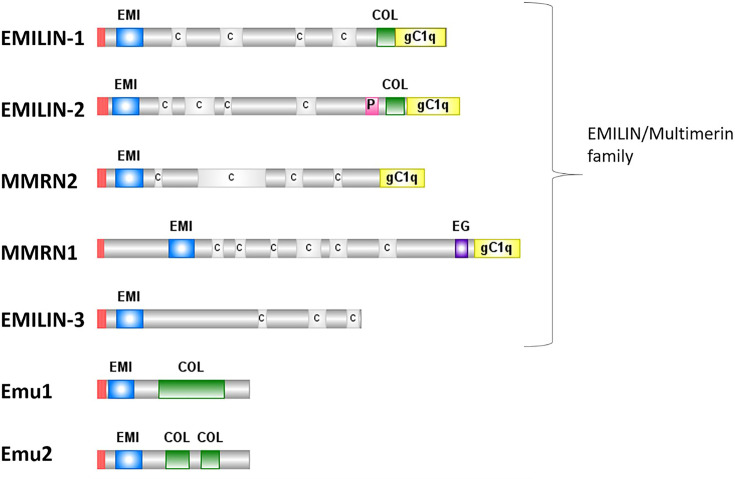
The protein domains of the EMILIN/multimerin family Emu1 and Emu2 are also shown, which together with the EMILIN/multimerin family have been proposed to form the EDEN superfamily. Red – signal peptide; blue ‘EMI’ – EMI domain; C – coiled-coil region; green ‘COL’ – collagen-like region; pink ‘P’ – proline-rich region; purple ‘EG’ – EGF-like domain.

**Figure 3:**
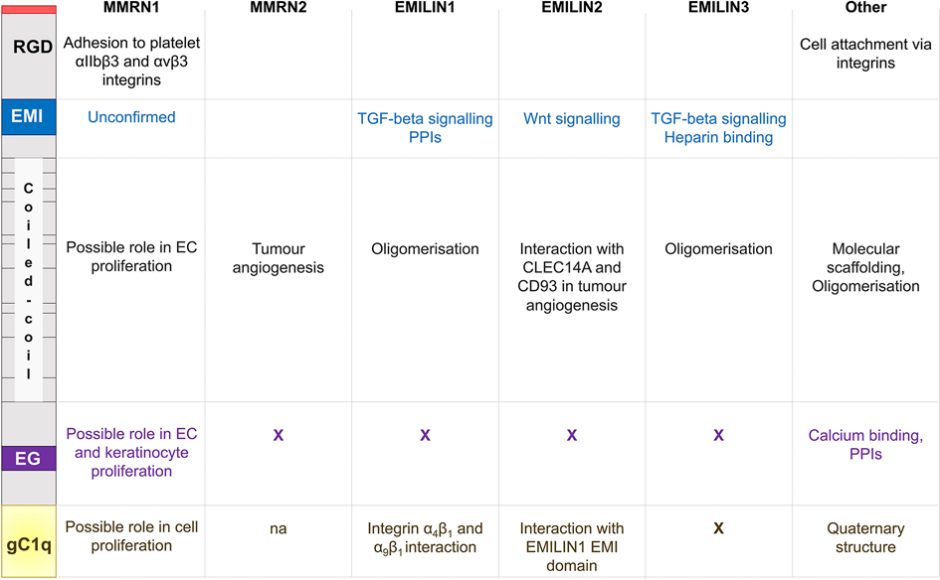
Known and proposed functions of protein domains found in the EMILIN/multimerin family The domains and RGD motif of MMRN1 are shown on the left and the reported functions for EMLIN/multimerin family members and other proteins are indicated. Abbreviation: PPI, protein–protein interaction.

MMRN1 is a large glycoprotein which contains an N-terminal EMI-domain, an epidermal growth factor (EGF)-like domain, coiled-coil, and a C-terminal gC1q domain [6,48] (Figure 2). Some domains are shared between different family members (EMI, gC1q) but their functions vary (Figure 3). The EMI domain of EMILIN-1 and EMILIN-3 has been shown to regulate TGF-β signalling [49,50], whereas in EMILIN-2 the EMI domain regulates Wnt signalling in breast cancer [31]. The EMI domain also mediates protein–protein interactions including the interaction between the EMILIN-1 EMI domain and the EMILN-2 gC1q domain [48,51], and heparin binding to EMILIN-3 [50]. The EMI domain of MMRN1 is unique amongst family members as it has six cysteine residues instead of seven [52]. If the MMRN1 EMI domain is also involved in protein–protein interactions and signalling events as its family members is unclear.

Within the EMILIN/multimerin protein family, the EGF-like domain is unique to MMRN1. This domain is associated with calcium binding, mediating protein–protein interactions [53] and is found in proteins linked to cell proliferation, differentiation and cancer, including various ECM proteins [54,55]. An MMRN1-derived peptide corresponding to 11 amino acids of the EGF-like domain has been shown to promote EC and keratinocyte proliferation *in vitro* [56], but this function is yet to be confirmed for MMRN1 *in vivo*.

Best known as the globular head domain of the complement protein C1q [57], the gC1q domain plays a role in protein multimerisation and quaternary structure formation [6,58–60]. The EMILIN1 gC1q domain interacts with integrins α_4_β_1_ and α_9_β_1_, regulating cell adhesion, lymphangiogenesis and tumorigenesis [61–64]. Interestingly, whereas the EMILIN gCq1 domain exerts an anti-proliferative effect, an MMRN1 gC1q-derived peptide has been reported to promote cell proliferation *in vitro* [56].

Common motifs in protein oligomerisation and macromolecular scaffolding [65], the coiled-coil domain is required for the oligomerisation of EMILIN1 [66] and EMILIN3 [50] and the same role has been inferred for MMRN1 and MMRN2. However, experimental evidence suggests additional roles of the coiled-coil region. Sheets et al. [56] showed that peptides corresponding to MMRN1 coiled-coil regions promoted EC proliferation *in vitro*. The interaction between the MMRN2 coiled-coil region and CLEC14A and CD93 plays a role in tumour angiogenesis [67].

The N-terminal RGD motif of MMRN1 is common to integrin-interacting ECM proteins and mediates cell adhesion. Integrin specificity, however, depends also on its conformational and spatial presentation [68]. MMRN1 binds to αIIbβ3 and αvβ3 integrins on activated platelets [5], but the interactions of the RGD motif of MMRN1 found in ECs and ECM with integrin has not been confirmed yet. However, adhesive interactions between tumour and ECs form part of the metastasis process and cell–cell interaction may be required for the endothelial transdifferentiation of metastatic melanoma cells to evade detection by the immune system [69]. When analyzing the interaction between melanoma and human umbilical vein endothelial cells (HUVECs), MMRN1 was one of the 30 upregulated genes with a possible role in cell–cell communication and tumour progression [70]. This may suggest a possible mechanism of MMRN1-mediated cell adhesion and melanoma-HUVECs communication. For example, MMRN2’s interaction with CD93 activates β1 integrin signalling, which leads to fibronectin fibrillogenesis and tumour angiogenesis [71]. Considering the importance of integrins in cancer [72], analysis of MMRN1–intergrin interactions in the context of ECs and ECM in cancer may reveal new MMRN1 interaction partners and functions. Such investigations will also confirm the role of proposed MMRN1 protein–protein interactions with serglycin [73,74], which is associated with poor prognosis of disease progression [75], and the oncogene *TC2N* [76]. The cell proliferative effect of various MMRN1 peptides indicates yet uncharacterised physiological roles of MMRN1 and will need further investigation.

## MMRN1 expression profile during development and in healthy tissue

In humans, MMRN1 is considered a marker of early neuroepithelium and is abundantly expressed in long-term self-renewing neuroepithelial-like stem cells [77] and primary osteoblasts [78]. MMRN1 is one of top 20 genes with specific expression in the adult lateral habenula [79]. There also appears to be a sex-specific expression pattern of MMRN1 in human ECs [80].

During mouse development, MMRN1 expression levels are only detectable in differentiated embryonic stem cells, including ECs lining blood vessels (perineural mesenchyme) and mesenchymal cells [81]. Whereas MMRN1 expression levels remain constant in most tissues throughout mouse development and following birth [81], MMRN1 levels increase during the course of murine erythroblast maturation [82]. Although MMRN1 protein levels have been reported to decrease with age [83], RNAseq of murine mammary epithelia and stroma suggests that the proportion and gene expression of lymphatic ECs, for which MMRN1 expression is a marker, remain fairly constant [84].

**Figure 4:**
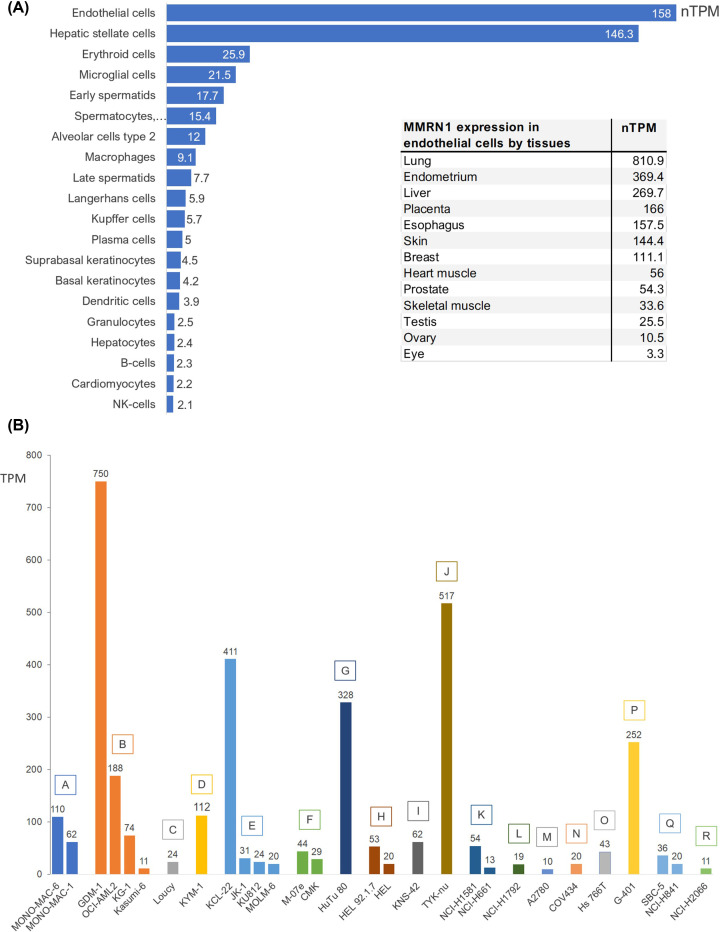
Expression of MMRN1 in cell types and cancer cell lines (**A**) The data show the expression of MMRN1 (nTPM > 2.0) in different healthy cell types. The expression data for these graphs were obtained from the Human Protein Atlas (https://www.proteinatlas.org/ENSG00000138722-MMRN1/single+cell+type) [85]. The inset shows the expression of MMRN1 in epithelial cells in specific organs/tissues. (**B**) Expression (TPM) of MMRN1 in various cancer cell lines representing adult acute monocytic leukaemia (A), adult acute myeloid leukaemia (B), adult T acute lymphoblastic leukaemia (C), alveolar rhabdomyosarcoma (D), blast phase chronic myelogenous leukaemia (BCR-ABL1 positive, E), childhood acute megakaryoblastic leukaemia (F), duodenal adenocarcinoma (G), erythroleukemia (H), glioblastoma (I), high-grade ovarian serous adenocarcinoma (J), large cell lung carcinoma (K), lung adenocarcinoma (L), ovarian endometrioid adenocarcinoma (M), ovarian granulosa cell tumour (N), pancreatic adenocarcinoma (O), rhabdoid tumour of the kidney (P), small cell lung carcinoma (Q), squamous cell lung carcinoma (R). The expression data were obtained from experiment E-MTAB-2770, EMBL-EBI Expression Atlas (Human Protein Atlas proteinatlas.org, [85]).

Human MMRN1 is predominantly expressed in ECs, especially in the lung (Figure 4A, data from Human Protein Atlas [85] (http://www.proteinatlas.org/)). It is an established EC marker [86] and *mmrn1* has been included in the group of ‘EC-restricted genes’ [87]. ECs show great heterogeneity, providing organ and tissue specific functions, and single-cell RNA sequencing (scRNA) of human lung ECs also identified MMRN1 expression in pulmonary–venous ECs [88]. MMRN1 also shows a distinct expression pattern in the lymphatic EC population in multiple human and murine organs and tissues (heart, muscle, lung, fat, lymph nodes, trachea, liver, middle ear, the eye) [89–95]. Interestingly, single-cell analysis of murine liver cancer identified a new lymphatic EC cluster, using *MMRN1* and *Pdpn* as marker genes [92]. These lymphatic ECs are associated with tumour tissue and have a different gene expression profile to blood vessel ECs, which may suggest a possible relationship with immune cells.

## MMRN1 differential expression in cancer

Transcriptome analysis of differentially expressed genes (DEGs) aids the identification of hub genes, biomarkers, classification of cancer subtypes and monitoring tumour progression [96–99]. Causes of altered gene expression can include gene mutations, deregulated transcription factors or the action of non-coding RNAs (ncRNAs), including microRNAs (miRNAs), long non-coding RNAs (lncRNAs) and circular RNAs (circRNAs), which can regulate each other (competitive endogenous RNA (ceRNA) hypothesis) [100,101].

**Figure 5:**
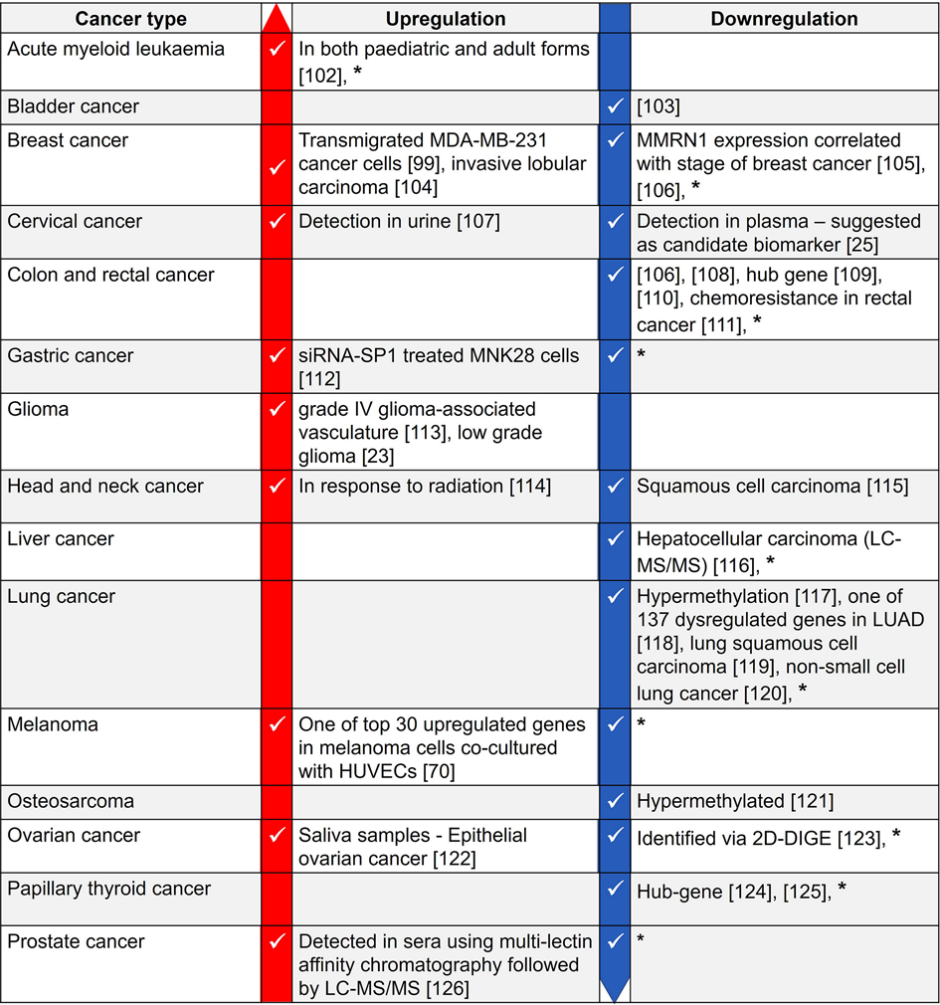
A summary of MMRN1 differential expression in various cancers The information is based on RNA-seq and proteomics data published in the stated references and databases. *: MMRN1 differential gene expression as per the TNM plot pan-cancer analysis [47].

MMRN1 is expressed in various cancer cell lines (Figure 4B), and a DEG in various cancers (Figure 5). It is a potential cancer biomarker in cervical cancer [25] and in paediatric acute myeloid leukaemia [24], where its expression is also positively correlated with the expression of the actin-binding protein Plastin 3 [127]. In rectal cancer, MMRN1 is one of five hub genes that act as prognostic biomarkers and elevated MMRN1 expression is associated with poor prognosis [109]. MMRN1 downregulation is associated with chemoresistance in rectal cancer [111] and radiosensitivity and associated clinical outcome in gastric cancer [128]. The *mmrn1* gene is associated with glaucoma [129] and bioinformatics analyses suggest *mmrn1* as a hub gene in papillary thyroid cancer [130].

In acute myeloid leukaemia, *mmrn1* is one of the genes that is upregulated by the action of the oncoprotein MLL-AF6, which is a fusion of the histone methyltransferase mixed lineage leukaemia (MLL) and the cytoplasmic protein AF6 as a result of gene rearrangement [131–133].

MMRN1 expression distinguishes leukaemia stem cells, which contribute to therapy resistance and disease relapse, from leukaemia progenitor cells [134,135]. In adult acute myeloid leukaemia, MMRN1 was identified alongside 16 other genes (*AKR1C3, ARHGAP22, CD34, CDK6, CPXM1, EMP1, GPR56, KIAA0125, LAPTM4B, NGFRAP1, NYNRIN, SMIM24, SOCS2, DNMT3B, DPYSL3, ZBTB46*), significantly correlated to leukaemia stemness and chemoresistance [102]. This finding led to the 17-gene leukaemia stem cell score (17LSC) which provides a prognostic measure of patient survival [102,136,137]. Similarly, MMRN1 expression levels alongside nine other mRNAs provide a prognostic risk score for gastric cancer patients [138], and Cai et al. [139] included MMRN1 expression in a risk score of papillary thyroid cancer. The observed correlation between MMRN1 expression and cancer risk scores highlights MMRN1 relevance in the disease process. That MMRN1 differential expression is cancer-specific has been shown in head and neck cancer, where MMRN1 downregulation is independent of Human Papilloma virus infections, which is often observed in this cancer type [115]. MMRN1 expression patterns have also been correlated with the stage of breast cancer [105], supporting its potential use in cancer diagnosis [24,139].

Considering the cancer-specific differential expression patterns of MMRN1 and possible application in cancer diagnosis, details on the regulatory mechanisms driving these expression patterns is relatively scarce. Below, we are discussing examples of potential regulatory mechanisms, including methylation, ncRNAs and transcription factors, which have been associated with MMRN1 expression levels in cancers.

## MMRN1 regulation via DNA methylation

Modulation of gene expression as a result of DNA methylation is commonly observed in cancer [140]. Although hypermethylation of MMRN1 has been reported in lung adenocarcinoma [117] and osteosarcoma [121], there appears to be no correlation with overall survival in lung cancer or the disease process in osteosarcoma. However, MMRN1 has a possible role in bone remodelling which may be linked to the observation in osteosarcoma [141,142].

## MMRN1 regulation via ncRNAs: miRNAs, lncRNAs, circRNAs

The role of miRNAs and lncRNAs in cancer is an ongoing area of research. miRNAs regulate gene expression by suppressing mRNA translation or degrading mRNAs, whereas lncRNAs are molecular scaffolds and form functional complexes with proteins and RNAs to regulate transcription [101,143–145]. ncRNAs are targeting MMRN1 in various cancers (Table 1). Wang et al. [146] showed that an increase in miRNA has-miR-374a (miR-374a), which has known oncogenic properties [147–149], downregulates MMRN1 expression in colorectal adenocarcinoma and colorectal cancer. miR-374a promotes cancer cell proliferation, migration and invasion by downregulating the tumour suppressor gene *SRCIN1* (also p140 cas-associated protein) in gastric cancer and regulates Wnt signalling pathways in non-small-cell lung cancer and breast cancer. In colorectal cancer, MMRN1 is targeted by has-miR-99b-5p [143], which is infrequently expressed in overall colorectal tumours, and its upregulation is associated with an increased likelihood of dying.

**Table 1:** Summary of known ncRNAs regulating MMRN1 in cancers

Cancer type	miRNAs	lncRNAs	circRNAs
**Colorectal cancer**	has-miR-374a has-miR-99b-5p		
**Acute myeloid leukaemia**		KIAA0125	
**Papillary thyroid cancer**	has-miR-4709-3p	LINC00506	
**Gastrointestinal stromal tumours**			hsa_circ_0070442

MMRN1 expression is positively correlated with higher expression of the lncRNA KIAA0125, which, like MMRN1, is included in the prognostic LSC17 for acute myeloid leukaemia [102] and is linked to a poorer prognosis, shorter overall survival and disease-free survival [150].

In papillary thyroid cancer, MMRN1 is co-expressed with the lncRNA LINC00506 but also targeted by the miRNA has-miR-4709-3p. The authors conclude that the interaction LINC00506–MMRN1–has-miR-4709-3p may be specific to this cancer [151].

circRNAs are ncRNAs derived from pre-mRNAs by alternative splicing. Their functions include sponging miRNAs and regulating gene expression and are associated with human diseases including cancer [152]. circRNAs are derived from the genetic information of a so-called ‘host gene’. In gastrointestinal stromal tumours, MMRN1 was identified as the host gene of the circRNA hsa_circ_0070442 and both are downregulated in this cancer. Interestingly, has_circ_0070442 is also one of the top 50 most downregulated circRNAs in lung squamous cell carcinoma [153], a cancer in which MMRN1 expression is also downregulated (based on data in TNM plot [47] and Oncomine [154]). However, a link between has_circ_0070442 and MMRN1 in lung squamous cell carcinoma has not been reported yet.

ceRNAs can be either mRNAs, lncRNAs, circRNAs or even pseudogene gene transcripts, which regulate each other by competitively binding to shared miRNAs [155]. Wen et al. [155] identified TRIB1 with MMRN1 as a ceRNA pair in breast cancer.

Regulation of MMRN1 by ncRNAs is not restricted to disease processes. An integrated transcriptome analysis identified has-miR-514a-3p, which targets MMRN1 in the ovulatory cascade [156].

## MMRN1 regulation by transcription factors

Transcription factors control gene expression and their deregulation is associated with cancer [157]. The transcription factor specificity protein 1 (SP1) is overexpressed in many cancers, including gastric cancer, and modulates cell proliferation and survival [158,159]. SP1 regulates MMRN1 expression *in vitro* in a gastric cancer model. MNK28 cells, which are well-differentiated stomach adenocarcinoma cells, have a high expression of SP1. Silencing SP1 in MNK28 cells with SP1 siRNA results in significant upregulation of MMRN1 (>2.5-fold) [112]. This aligns with the observation that in gastric cancer, where SP1 levels are high, MMRN1 levels are downregulated (Figure 5).

Most information on possible transcription factors that target MMRN1 in various cancers is currently available through bioinformatics analyses and offer an interesting starting point for future in *vitro* and *in vivo* experiments. Network analyses identified the transcription factors SP1 and NFIC as regulators of MMRN1 in lung adenocarcinoma [160] and lung squamous cell carcinoma respectively [119]. An association between transcription factor FOX3A and MMRN1 has been reported in anaplastic thyroid carcinoma [161]. The transcription factor-binding sites for ETS-FOXC2 and ETS are present in the *mmrn1* gene [162], but there is currently no information if these are targeted to regulate MMRN1 expression in cancers.

## MMRN1 alternative splicing and copy number alterations in cancer

The *mmrn1* gene is located on chromosome 4 (4q22.1) and contains 11 distinct introns which code for six different isoforms [163]; exon skip events for platelet MMRN1 have been reported previously [164]. Transcriptome analysis of colorectal adenocarcinoma samples identified MMRN1 as one of the 206 genes involved in alternative splicing events in colorectal cancer [165]. In this study, other genes coding for proteins with a role in cell adhesion were identified, but of the original 206 genes, only 18 gene candidates (not including MMRN1) were considered as relevant to cancer development. At the point of writing this review, no other evidence of *mmrn1* alternative splicing events in cancer or another disease process was found in the literature.

In non-germinomatous malignant germ cell tumours (NGMGCTs), which are a type of intracranial paediatric germ cell tumour, the *mmrn1* gene is deleted due to copy number variations at cytobands 4q13.3-4q28.3 [166]. Studying BIN-67 cells, a model for the rare and aggressive form of small cell ovarian carcinoma of the hypercalcaemic type, SNP array analyses identified copy number loss of 4q22.1, which entails the SNCA and MMRN1 gene [167]. BIN-67 cells are resistant to conventional chemotherapeutics and NGMGCTs are less perceptive to drug and radiation treatment than other germ cell tumours [166]. Although there is no clear involvement of MMRN1 in these cancers on the basis of the studies, it is interesting that these cancers are less responsive to treatment as are other cancers with differential MMRN1 expression e.g., rectal cancer [111].

## The effect of signalling cascades and hormones on MMRN1 expression

MMRN1 was classified as an immune-related gene that was only expressed in glioblastoma with high interleukin-13 receptor α2 mRNA expression [168]. Dieterich et al. [113] reported MMRN1 as one of the 78 upregulated genes in the vasculature of grade IV glioma in response to increased VEGF-A and TGFβ2 signalling in the tumour microenvironment. This suggests a possible role of MMRN1 and the other upregulated genes in angiogenesis and VEGFR signalling which are involved in tumorigenesis and metastasis [169].

Hormones like oestrogen and progestogens contribute to breast cancer risk [170,171] and oestrogen can affect gene expression patterns [172,173], including MMRN1. Pitteri et al. [174] reported an increase in serum MMRN1 protein concentration in healthy postmenopausal women who had taken oestrogen and progestin (an exogenous synthetic progestogen) versus oestrogen only. MMRN1 mRNA levels were upregulated in both endometrial stromal cells treated with β-oestradiol [175] and in the endometrium in response to a high serum progesterone level [176]. Whereas MMRN1 levels appear high in these healthy conditions, MMRN1 levels are downregulated in the majority of reported breast cancer datasets (Figure 5). Considering the role of hormones in types of breast cancer, it would be interesting to investigate if MMRN1 is hormonally regulated in the future.

## MMRN1 protein detection in cancer

Most of the data of MMRN1 in cancer are based on transcriptional analysis, but MMRN1 protein levels in bodily fluids are being explored in cancer diagnostics Although MMRN1 is considered undetectable in plasma [5], MMRN1 levels have been detected in the plasma, serum, urine and saliva of cancer patients [107,122,123]. Saini et al. [122] report detection of MMRN1 in the saliva of epithelial ovarian cancer patients but comment that the route by which MMRN1 enters the saliva remains unclear. Vizecoumar et al. [177] identified *MMRN1* as a downregulated gene whose protein product is detectable in the plasma in gastro-oesophageal cancer patients. Using mass spectrometry techniques, changes in MMRN1 protein levels were detected in the sera of multiple myeloma patients [178] and hepatocellular carcinoma patients [116] and in cell lysates from bone marrow aspirates from patients with amyloid leukaemia [179]. Multi-lectin chromatography, followed by LC-MS/MS identified elevated MMRN1 protein levels in sera from patients with prostate cancer and benign prostate hyperplasia [126]. SWATH-MS identified a positive correlation between MMRN1 and thrombospondin 1 expression, which is differentially regulated in cancers, in the blood plasma of five cancers (colorectal, pancreatic, lung, prostate, ovarian) [180]. Perhaps MMRN1’s presence in various bodily fluids is not entirely a surprise. It has been postulated that proteins are secreted or shed from cancer tissues [180] and it has been shown that MMRN1 is being released via exosomes by duodenal cancer cells [181] (for MMRN1 expression in duodenum adenocarcinoma cell line HuTu80, see Figure 4B), bladder cancer cells [182], and medulloblastoma cells [183]. Exosomes released by UM-SCC6 head-and-neck cancer cells which were treated with ionising radiation had upregulated MMRN1 levels [114]. Exosomes are able to promote metastasis [184], but how MMRN1 exosomal release is associated with cancer progression has not been investigated yet. Elevation of MMRN1 levels in serum exosomes has also been observed in burn patients [185] and patients with tuberculosis infection [186].

## Future directions on the functional roles of MMRN1

MMRN1’s potential as a biomarker in certain cancers, including diagnosis of cancer stages, is a strong possibility [23–25,105]. However, detail on MMRN1 protein interactions and involvement in signalling events is required to describe MMRN1 molecular mechanisms and if and how this correlates with its differential expression. If MMRN1 is to be used for cancer diagnosis, detection methods need to be optimised and standardised to provide reliable MMRN1 detection, which can be carried out in a clinical/diagnostic setting e.g., in cervical cancer MMRN1 expression is observed to be either up- or downregulated, depending on whether urine [107] or plasma [25] was analysed (Figure 5). This opposing trend does not exclude MMRN1’s feasibility as biomarker; MMRN1 protein is specifically detected in ovarian cancer [187]. Instead, it highlights the lack of detail to explain MMRN1 protein levels in different bodily fluids and why they vary with disease. For example, MMRN1 has notable mRNA levels in the cervix and cervical mucus, which is a known component of first-void urine samples [188] which may explain MMRN1 detection in urine, but this will need to be verified to support the use MMRN1 as a biomarker in cervical cancer.

Currently, it is difficult to confirm any specific MMRN1 role that may aid or hinder cancer progression, but MMRN1 downregulation in non-small-cell lung cancer has been hypothesised to contribute to vessel leakage and poor blood vessel repair, which would facilitate access of oxygen and nutrients to cancer cells [120]. The *in vitro* data on MMRN1 peptides corresponding to EGF, gC1q and coiled-coil regions in promoting cell proliferation [56], as well as MMRN1’s potential role in cell adhesion and cell–cell communication [70], certainly warrant further investigations into MMRN1 physiological roles in cancer. Therefore, biochemical and structural studies into MMRN1’s mechanistic roles are needed and may provide support for MMRN1’s use in cancer diagnosis and prognosis.

The role of MMRN1’s cellular localisation is also little understood. Platelets, ECs and the ECM play key roles in cancer. Cross-talk between platelets and tumour cells drives cancer development and progression [41,189] and can facilitate metastasis of solid tumours [40,190]. Malignant tumours also stimulate platelet production (paraneoplastic thrombocytosis), and high platelet numbers correlated with poor cancer prognosis [41,191]. The ECM is remodelled by tumours to create optimal tumorigenic conditions [192]. ECs are embedded in the ECM, both forming part of the tumour microenvironment. Tumour cells regulate ECs to induce processes like angiogenesis, which support their growth and the process of endothelial-to-mesenchymal transition is involved in tumour progression [193]. High-resolution microscopy, proteomics and protein interaction studies can provide information on MMRN1 protein distribution, abundance and interactions in health and disease states and help characterise MMRN1 function in different cellular localisations.

In addition to cancer, MMRN1 is differentially expressed in other diseases including inflammation and bacterial and viral infections [186,194–197]. Upregulation of MMRN1 has also been observed in injuries including septic shock-associated kidney injury [198] and in serum exosomes from burn patients [185]. MMRN1 may also play a role in human pathogen interactions. The MMRN1 protein is the target of *Staphylococcus aureus* extracellular fibrinogen binding protein (Efb) [199], the *Helicobacter pylori* vacuolating cytotoxin VacA [200], and MMRN1-derived peptides inhibit *Streptococcus pneumoniae* adhesion to ECs [201].

The current information indicates that MMRN1 is involved in various disease states that are of medical interest. Transcriptome and bioinformatics analyses have provided the evidence on MMRN1 differential expression. In the next step, characterisation of MMRN1’s physiological functions and molecular mechanisms in ECs and the ECM will be necessary to identify new diagnostic and treatment strategies.

## Abbreviations

ceRNA: competitive endogenous RNA
circRNA: circular RNA
DEG: differentially expressed gene
EC: endothelial cell
ECM: extracellular matrix
EGF: epidermal growth factor
HUVEC: human umbilical vein endothelial cell
lncRNA: long non-coding RNA
miRNA: microRNA
MMRN1: multimerin-1
ncRNA: non-coding RNA
NFIC: nuclear factor 1 C-type
NGMGCT: non-germinomatous malignant germ cell tumour
RGD motif: arginine-glycine-aspartic acid motif
SP1: specificity protein 1
VEGF: vascular endothelial growth factor
